# Hippocampal Neurogenesis and the Brain Repair Response to Brief Stereotaxic Insertion of a Microneedle

**DOI:** 10.1155/2013/205878

**Published:** 2013-03-11

**Authors:** Shijie Song, Shuojing Song, Chuanhai Cao, Xiaoyang Lin, Kunyu Li, Vasyl Sava, Juan Sanchez-Ramos

**Affiliations:** ^1^Department of Neurology, University of South Florida, 13220 Laurel Drive, Tampa, FL 33612, USA; ^2^Research Service, James A. Haley VA Medical Center, Tampa, FL 33612, USA; ^3^Feinberg School of Medicine, Northwestern University, Chicago, IL 60611, USA; ^4^Department of Molecular Pharmacology and Physiology, University of South Florida, Tampa, FL 33612, USA

## Abstract

We tested the hypothesis that transient microinjury to the brain elicits cellular and humoral responses that stimulate hippocampal neurogenesis. Brief stereotaxic insertion and removal of a microneedle into the right hippocampus resulted in (a) significantly increased expression of granulocyte-colony stimulating factor (G-CSF), the chemokine MIP-1a, and the proinflammatory cytokine IL12p40; (b) pronounced activation of microglia and astrocytes; and (c) increase in hippocampal neurogenesis. This study describes immediate and early humoral and cellular mechanisms of the brain's response to microinjury that will be useful for the investigation of potential neuroprotective and deleterious effects of deep brain stimulation in various neuropsychiatric disorders.

## 1. Background 

Deep brain stimulation through chronically implanted metal electrodes into specific brain regions is becoming a common therapeutic choice for medication refractory movement disorders such as Parkinson's disease (PD), tremors, and dystonia (see reviews [1–3]). More recently, DBS has been applied to psychiatric and behavioral disorders including depression, obsessive compulsive disorder, and addiction and most recently to disorders of consciousness [4–9].

Long-term implantation of a fine metal electrode, even without chronic electrical stimulation may produce unwanted effects. Neuropathological examination of brain tissue from patients with DBS revealed activated astrocytes and microglia regardless of the underlying disease [10–15]. Electrical stimulation is not required to see signs of neuroinflammation; inflammatory changes have been observed around recording electrodes used for characterizing epileptogenic tissue and around CSF fluid shunt catheters [16, 17].

 To understand the earliest reactions to implantation of a metal electrode, we studied the cellular and cytokine responses over time to transient insertion of a fine needle (maximum diameter of 200 *μ*m) into the dorsal hippocampus of the mouse. We tested the hypothesis that the creation of a focal microlesion in hippocampus elicits self-repair mechanisms mediated by cytokines which activate microglia, promote astrocytosis, and stimulate stem/progenitor cells to proliferate and generate new neurons.

## 2. Materials and Methods

All procedures described here were reviewed and approved by the IACUC Committee of the University of South Florida and the Haley VA Research Service.

### 2.1. Animals

C57BL/6 mice, 8–10 weeks old, were purchased from Harlan Laboratories, and transgenic GFP mice (C57BL/6-Tg [ACTB-EGFP] 1Osb/J, 003291) were purchased from Jackson Laboratory (Bar Harbor, ME). Most of the experiments utilized groups of C57BL/6 mice, and one experiment utilized chimeric mice (C57BL/6 mice transplanted with green fluorescent protein expressing (GFP+) bone marrow).

### 2.2. Generation of Chimeric Mice

The procedure for bone marrow harvesting from tg GFP+ mice has been previously published by Sanchez-Ramos and coworkers [18, 19]. Briefly, bone marrow cells are collected from femurs and tibias of adult male GFP transgenic mice by flushing the bone shaft with PBS + 0.5% bovine serum albumin (BSA) + 2 mM ethylenediaminetetraacetic acid (EDTA) (Sigma).

To generate chimeric mice, C57BL/6J mice were lethally irradiated with 8 Gy total body irradiation (delivered in two fractions of 4 Gy, an interval of 4 hours) at dose rate of 1.03 Gy/min in a Gammacell 40 Extractor [20]. Following irradiation, the mice were given a bone marrow transplant (10 × 10^6^ mononuclear cells) from transgenic GFP mice infused via tail vein. Bone marrow-derived cells in the rescued mice were readily tracked by virtue of their green fluorescence. Examination of blood smears from tail clippings for the presence of green monocytes confirmed successful engraftment.

### 2.3. Stereotaxic Insertion and Removal of Microneedle

Animals were anesthetized with sodium pentobarbital (50 mg/kg, i.p.) and placed into a stereotactic frame. Using bregma as the reference point, a trephine hole was then drilled in the skull and the needle was gently inserted into the hippocampus (AP 2.5 mm; ML 1.3 mm; DV 3.5 mm). Mice received 5-bromo-2′-deoxyuridine (BrdU) (Sigma) injections (100 mg/kg i.p. Bid, immediately after the surgery and 2 days after surgery) to label nascent cells during a 3-day period.

### 2.4. Tissue Preparation and Sectioning

At one, two, and four weeks after needle stimulation, mice were anesthetized with 10% chloral hydrate and a transcardial perfusion of the brain with 20 mL saline and 50 mL of 4% paraformaldehyde was done. The brain was removed and fixed for 48 hours in the same solution. After fixing, the brains were immersed overnight in 20% sucrose in PBS. Thirty *μ*m frozen sections through the striatum, hippocampus, midbrain, and cerebellum were prepared and stored in vials containing a cryopreservation solution.

### 2.5. Immunohistochemistry

Brain sections were preincubated in PBS containing 10% normal serum (goat or donkey; Vector) and 0.3% Triton X-100 (Sigma) for 30 min. The sections were then transferred to a solution containing primary antibodies in 1% normal serum and 0.3% triton X-100/PBS and incubated overnight at 4°C. The specific antibodies used in each experiment were rat anti-BrdU (Serotec), 1 : 100; mouse anti-NeuN (Chemicon), 1 : 50; mouse antinestin (BD Biosciences); rabbit anti-DCX (Abcam Inc.), 1 : 1000; rabbit anti-Iba1(Wako Chemicals, USA, Inc.), 1 : 500; rabbit anti-GFAP (BioGenex), 1 : 50 in PBS containing 1 : 100 normal serum without Triton X-100. After incubation with primary antibody, the sections were washed and incubated for 1 hour with Alexa Fluor 488 goat anti-mouse IgG diluted 1 : 400 in PBS or Alexa Fluor 546 goat anti-rabbit IgG diluted 1 : 600 in PBS (Invitrogen) at room temperature. Isotype controls matching the primary antibody's host species (mouse) were used in place of the primary antibody (monoclonal to NeuN and Nestin) to check for specificity of the stain. The sections were then rinsed in PBS three times and covered with a cover glass. Some sections were stained (after all other staining) with DAPI (300 nM) for nuclear staining. Fluorescent signals from the labeled cells were visualized with fluorescence microscopy using appropriate filters or a Zeiss LSM510 confocal microscope.

### 2.6. Image Analysis and Cell Counts

Quantitation of microgliosis and astrogliosis was made by computerized image analysis. Images at 20x magnification were acquired as digitized tagged-image format files to retain maximum resolution using an Olympus BX60 microscope with an attached digital camera system (DP-70, Olympus). Digital images were routed into a Windows PC for quantitative analyses using ImageJ software (NIH). Images of six sections (180 *μ*m apart) were captured from serially sectioned hippocampus. Color images were separated into green, red, and blue channels. The monochrome image for green (either Iba1 or GFAP) was then processed by setting a threshold to discriminate staining from background. Each field of interest was manually edited to eliminate artifacts. For the Iba1 (microgliosis) and GFAP (astrocytosis) burden analyses, data are reported as the percentage of labeled area captured (positive pixels) divided by the full area captured (total pixels). Bias was eliminated by analyzing each entire region of interest represented by the sampling of 6 sections per hippocampus. A total of 6–8 mice hippocampi were analyzed. 

 Unbiased estimates of the number of immature neurons dentate gyrus (DG) were made by counting DCX-immunoreactive cells in serially sectioned hippocampus according to the method previously described [21, 22]. Estimates of numbers of BrdU labeled microglia (Iba1-BrdU+) cells in hippocampus were also determined. Briefly, positively labeled cells were counted in every 6th section (each section separated by 180 *μ*m) using a modification to the optical dissector method; cells on the upper and lower planes were not counted to avoid counting partial cells. The number of DCX+ cells counted in every 6th section was multiplied by 6 to get the total number of DCX cells in the DG and Iba1 cells in hippocampus. The total number of hippocampi analyzed was 3 for each time period. The unlesioned left hippocampus served as control.

### 2.7. Cytokine Assay

After creating a right-side hippocampus microlesion, mice were euthanized at 6, 12, 24, 48, and 72 hours (*n* = 3 mice per time interval) followed by perfusion with saline. Frontal cortex and hippocampus of the left and right brains were dissected and kept in freezer for cytokine assay. Levels of 17 cytokines were measured using Bio-Rad Bio-Plex kits (Bio-Rad, catalogue number 171F11181). Samples and standards were prepared using company protocols with the initial concentration of standards ranging from 32 ng/mL to 1.95 pg/mL. Samples were prepared for analysis by diluting 1 volume of the tissue sample with three volumes of the Bio-Plex mouse sample diluent. Using the microplate readout, each cytokine level was calculated based on its own standard curve.

### 2.8. Statistical Analysis

Neurohistologic measures were expressed as mean ± SEM and statistically evaluated using 2-way ANOVA followed by Bonferroni corrections for multiple comparisons (GraphPad version 5.01). The time course of cytokines release was analyzed using 2-way ANOVA. All comparisons were considered significant at *P* < 0.05.

## 3. Results 

**Figure 1:**
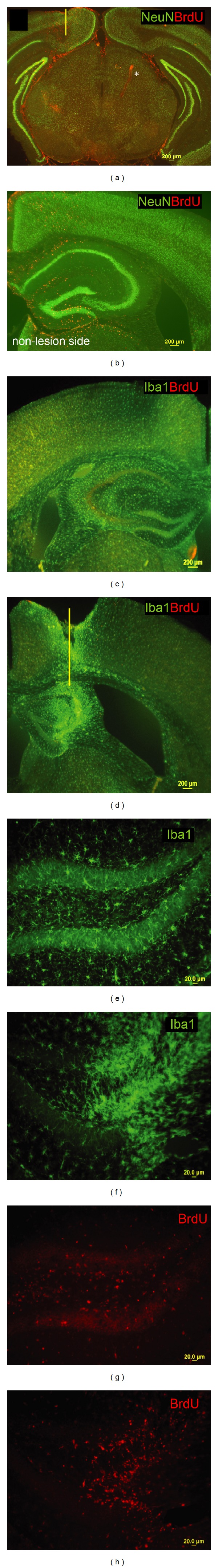
Cellular response to insertion and removal of a microneedle. (a) Low power view of cells labeled with BrdU in region of hippocampus and midbrain (BrdU = red; NeuN = green); needle was inserted on the right side of brain (yellow line). BrdU injections were given on the day of lesion and subsequent two days. Image taken one week after lesion. BrdU+ cells are found along the needle track and in the subarachnoid space and vasculature on both sides of brain. Scale bar = 200 *μ*m for panels (a), (b), (c), and (d). (b) Hippocampus (rostral to section in (a)) from the same animal on the unlesioned side. BrdU+ cells are seen in cortex, corpus callosum, the subarachnoid space, dentate gyrus, subgranular zone, and stratum lacunosum molecular of the hippocampus (one week after lesion). (c) Nonlesioned hippocampus opposite the lesioned hippocampus in panel (d) (2 wks after lesion). (d) Site and track of needle insertion (yellow line). Two weeks after lesion. (e) Nonlesioned hippocampus at higher power (Iba1 = green scale; bar = 20 *μ*m for panels (e), (f), (g), and (h)). (f) Iba1+ cells in lesioned hippocampus. (g) BrdU+ cells on nonlesioned side, corresponding to panel (e). (h) BrdU+ cells on lesioned side, corresponding to panel (f).

 Insertion and immediate removal of a fine needle to the hippocampus on one side of brain resulted in mobilization of cells along the needle track. BrdU+ cells labeled *in vivo* during the 3 days after placement of the lesion were found along the microinjury track through cerebral cortex to the hippocampus and to a lesser extent were observed along the corpus callosum on both sides of the brain (Figure 1). Although many of the BrdU+ cells appear to be derived from peripheral blood, any cell with proliferative capacity within brain and its lining membranes were also labeled. BrdU+ cells were found in the needle-breeched subarachnoid space and cerebrospinal fluid (CSF), from where they have access to hippocampus by way of the CA3-dentate gyral border with the ventricle, even on the nonlesioned side (Figure 1(b)).

**Figure 2:**
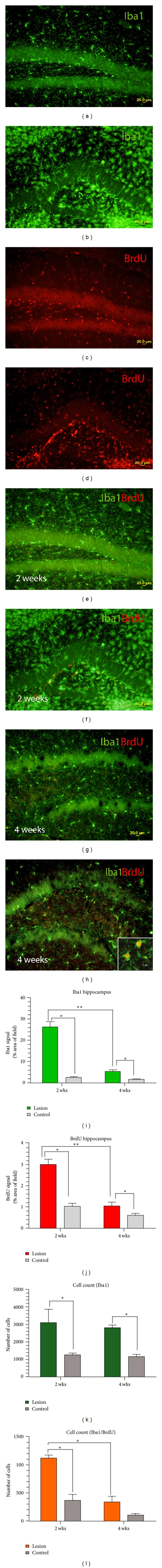
Microgliosis indicated by Iba1 immunostaining in hippocampus at 2 wks and 4 wks after microlesion. Panels on the left ((a), (c), (e), and (g)) illustrate the microglial response on the unlesioned control side, and the panels on the right ((b), (d), (f), and (h)) are the corresponding lesioned sides. Panels (a), (b) = Iba1 immunostaining; (c), (d) = BrdU+ cells corresponding to sections (a) and (b), respectively. Panels (e), (f) are merged images of Iba1-BrdU signal 2 wks after lesion. Panels (g), (h) are merged images of Iba1-BrdU signals 4 wks after lesion. Insert box shows a merged confocal microscopic image of double-labeled Iba-1-BrdU. (i) Microgliosis assessed by image analysis of Iba1 signal. *y*-axis = mean Iba1 signal area (as percent of hippocampal field at 20x magnification). Microglial signal was 16 times greater on the lesioned side than control at 2 wks (*P < 0.001). The microglial signal on the lesioned side declined significantly after 4 wks (**P < 0.001) but remained significantly elevated compared to the unlesioned side. (j) BrdU signal area was 3 times greater on the lesioned side at 2 wks and declined after 4 wks. (k) A number of Iba1+ cells were approximately 2.45 times greater on the lesioned hippocampus compared to the nonlesioned control at both 2 and 4 wks (*P < 0.01). Notice that microglia in the lesioned hippocampus are larger than on the control side, and therefore, the total Iba1 signal area is much greater than total number of cells. (l) A number of double-labeled microglia (Iba1/BrdU) were also greater on the lesioned side than controls at both 2 wks (*P < 0.05). Double-labeled microglia comprised ~36% of the total number of Iba1+ cells. Scale bar = 20 *μ*m.

The labeling of tissue sections with anti-Iba1 antibodies revealed both a significant proliferation and enlargement of microglial cells (Figure 2). Two weeks after placement of the lesion, the mean Iba1 signal area per field, reflecting both size and number of cells, was 16 times the signal on the unlesioned control side (Figures 2(a), 2(b), and 2(i)). At four weeks, the signal on the lesioned side remained elevated but was decreased compared to the signal at 2 wks, suggesting a time-dependent downregulation of microgliosis in this model (Figures 2(h) and 2(i)). The mean BrdU signal area was 3 times greater on the lesioned side than the control side, but like the Iba1 signal, BrdU area decreased significantly from 2 wks to 4 wks (Figure 2(j)). The number of Iba1+ cells was approximately 2.45 times greater on the lesioned hippocampus compared to the nonlesioned control at both 2 and 4 wks (Figure 2(k)). The microglia in the lesioned hippocampus were morphologically larger than on the control side, and therefore the total Iba1 signal area is much greater than the total number of counted cells. The Iba1+ cell counts likely underestimated the true number of microglia because individual cells were difficult to distinguish in regions where the intense microgliosis resulted in clusters of Iba1+ staining. However, when Iba1+ cells that had a nucleus labeled with BrdU (Iba1/BrdU) were counted, there was clearly a significantly greater number of Iba1/BrdU+ cells on the lesioned side than in controls at 2 wks (*P < 0.05). Double-labeled microglia comprised ~36% of the total number of Iba1+ cells, suggesting that a significant proportion of microglia were born after the 3 days of labeling with BrdU.

**Figure 3:**
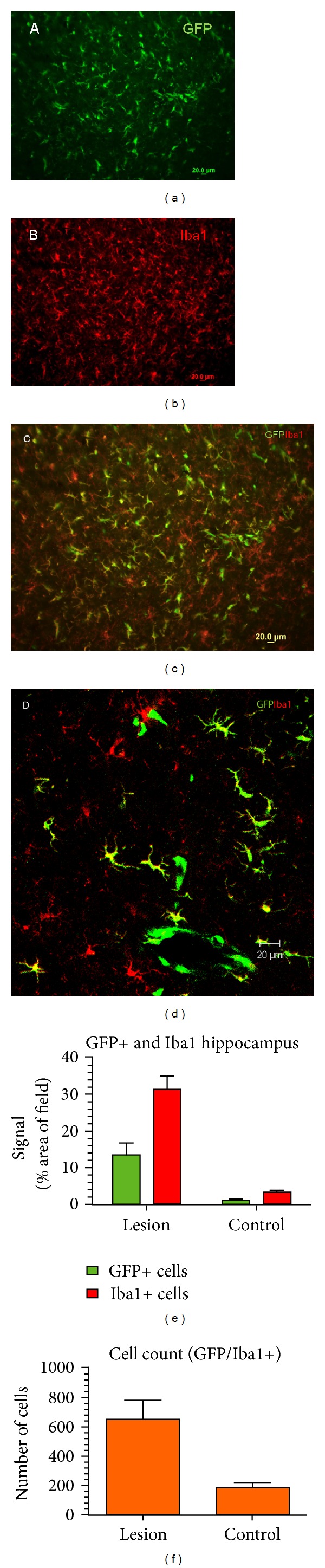
Contribution of blood-derived cells to microgliosis in chimeric mice 2 weeks after lesion placement. (a) Blood-derived GFP+ cells in hippocampal region. (b) Microgliosis two weeks after the lesion is indicated by Iba1 immunoreactivity. (c) Merged images of GFP+ (A) and Iba1+ cells (B). (d) Merged confocal image showing double-labeled microglia (yellow) at a higher magnification. (e) Mean Iba1 and GFP+ signals. On the lesioned side, the mean GFP+ signal is 26% of the total Iba1 signal (ratio of 13.4/51.6). (f) Cells count of double-labeled Iba1/GFP cells is significantly greater on the lesioned side than control.

 The contribution of blood-derived cells (GFP+ cells in chimeric mice) to the total microglial population is shown in Figure 3. The image analysis of GFP+ and Iba1 signals on the lesioned side revealed a mean GFP+ signal equal to 26% of the total Iba1 signal (ratio of 13.4/51.6), suggesting that approximately one-fourth of the microglial signal comes from the peripheral blood (GFP+ bone marrow-derived cells). Cells counts of double-labeled Iba1/GFP cells confirm a significantly increased number of blood-derived microglia on the lesioned side compared to the unlesioned side. 

**Figure 4:**
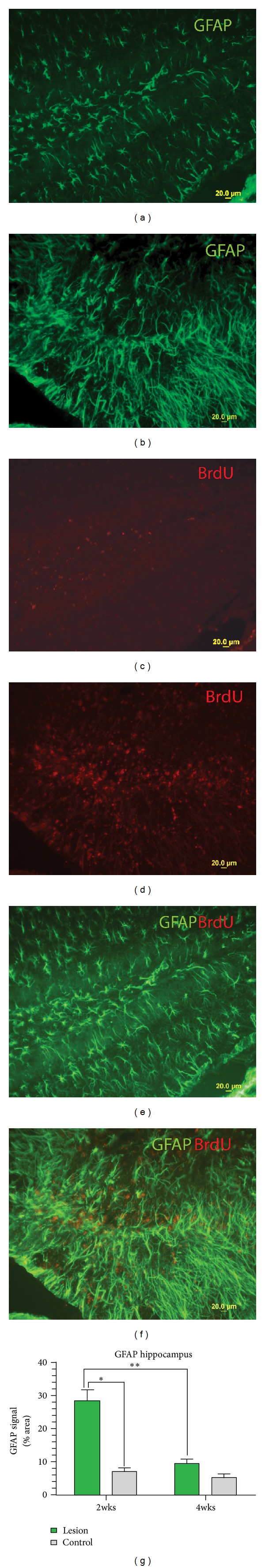
Astrocytosis in hippocampus indicated by GFAP immunoreactivity. Panels on the left ((a), (c), and (e)) depict the unlesioned control side; panels (b), (d), and (f) show the lesioned side. (a), (b) = GFAP (green channel); (c), (d) = BrdU (red channel); (e), (f) merged channels (GFAP and BrdU). (g) Astrocytosis measured as extent of GFAP immunoreactivity (mean % area of field) was increased 6 times that of control (*n* = 3 mice at 2 wks; 3 mice at 4 wks; 6 sections per mouse). 2-way ANOVA showed that both treatment and time contribute significantly to the variance. Signal was significantly higher on lesioned side at 2 weeks. At 4 weeks, the mean signal had declined significantly. *P < 0.001; **P < 0.001 using Bonferroni correction for multiple comparisons.

 The microlesion also triggered significant astrocytosis, indicated by GFAP immunoreactivity (Figure 4). GFAP signal on the lesioned side was 6 times that of the nonlesioned control side. Like the microgliosis, the GFAP signal decreased by 4 wks after the lesion (Figure 4(g)). Counts of GFAP+ cells were not done because of difficulty in discerning individual GFAP+ astrocytes in many regions of astrocytosis.

**Figure 5:**
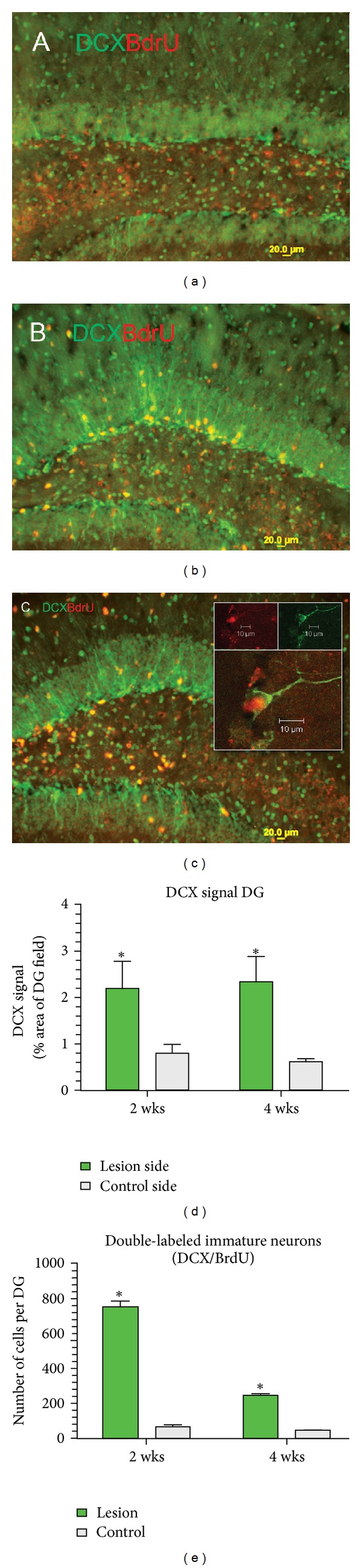
Microlesion stimulates neurogenesis. (a) Merged image of DCX and BrdU immunoreactive cells on the unlesioned control side (2 wks after the lesion). (b) Lesioned side illustrates increased DCX and BrdU (merged image). (c) Same as panel (b), but magnified; scale bar = 20 *μ*m. Doublecortin (DCX) Immunoreactive cells in the subgranular zone of the dentate gyrus extend processes into the granular zone. The box inserted in (c) depicts confocal images of double-labeled DCX-BrdU cell at a higher power. Upper two panels are isolated for DCX (green) and BrdU (red) immunofluorescence, and the lower panel is the merged image (scale  bar = 10 *μ*m). (d) Summary data of DCX signal expressed as percent of DG field. Lesioned side exhibits a significantly increased DCX signal compared to control at both 2 and 4 wks after the microlesion. Unlike microgliosis and astrocytosis, the DCX signal does not decline after 4 wks. (e) Cell counts of double-labeled immature neurons (DCX/BrdU) born within 2 days of lesion placement. The lesion significantly increased birth of new neurons compared to unlesioned control side. *P* < 0.001. However, the number of double-labeled cells was significantly less at 4 wks than observed at 2 wks.

 Insertion and removal of the needle stimulated neurogenesis in the subgranular zone of hippocampus, indicated by immunostaining for doublecortin (DCX), a marker of immature neurons (Figure 5). The mean DCX signal in dentate gyrus was significantly increased at 2 weeks and remained increased at 4 wks compared to the nonlesioned control side (Figure 5(d)). Unbiased estimates of cell counts of DCX-BrdU colabeled cells were also increased significantly at 2 and 4 weeks (Figure 5(e)). The total number of double-labeled cells was diminished at 4 wks compared to 2 wks, suggesting that many new neurons, born in the immediate days after lesion placement, undergo subsequent apoptosis. However, DCX+ cells, unlabeled with BrdU, were clearly maintained at approximately the same level at 2 and 4 wks, suggesting there are cytokine signals that continue to stimulate generation of new neurons beyond the time frame of BrdU injections (i.e., first 3 days of microlesioning).

**Figure 6:**
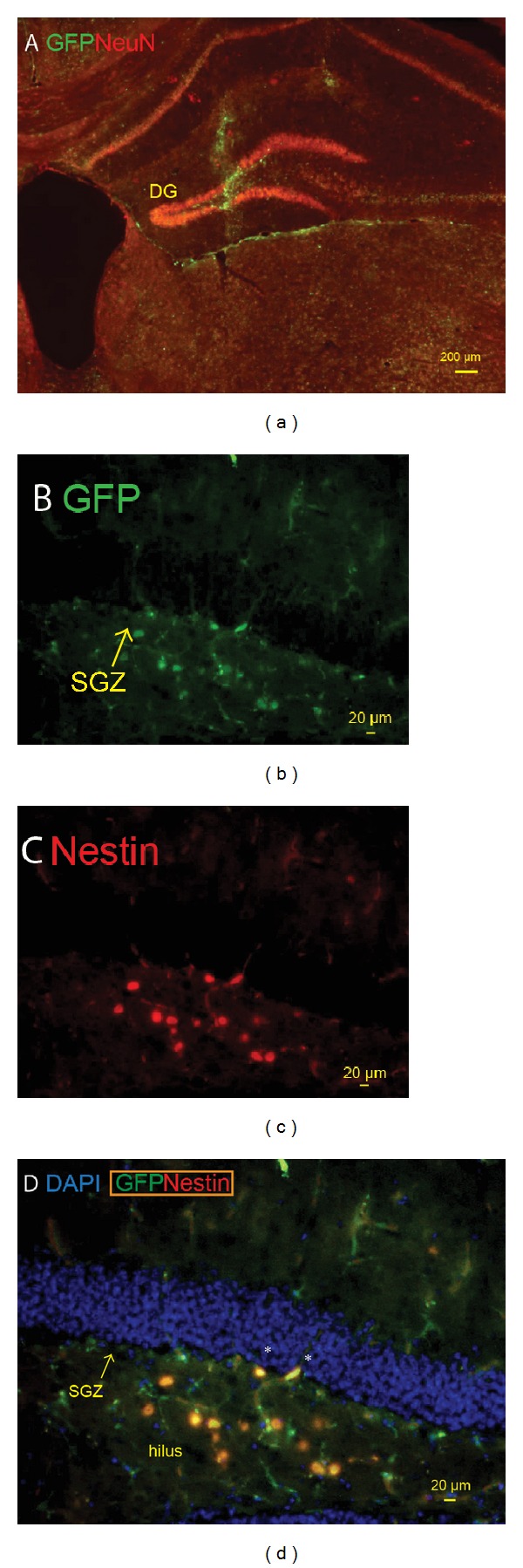
Chimeric mice stained for GFP (green) and Nestin (red). (a) GFP+ cells can be seen along the needle track and infiltrating in the sub-granular zone (SGZ) of the dentate gyrus (DG) and the CSF fluid space ventral to the hippocampus (scale bar = 200 *μ*m). (b) GFP+ cells in the SGZ of the DG. (c) Nestin+ cells in SGZ of the DG. Most of the GFP+ cells are in the hilus (also known as CA4). (d) Merged image illustrating coexpression of GFP and nestin in two cells (asterisks) in the immediate SGZ (scale bar = 20 *μ*m).

 The contribution of blood-born GFP+ cells to increased neurogenesis was examined (Figure 6). Rare GFP+ cells were found to coexpress nestin in the neurogenic niche (Figure 6(d)) but these cells did not express the typical fibrillary processes of neural progenitors in the subgranular zon [23]. Hence, increased neurogenesis triggered by the lesion could not be attributed to transdifferentiation of blood-derived cells.

**Figure 7:**
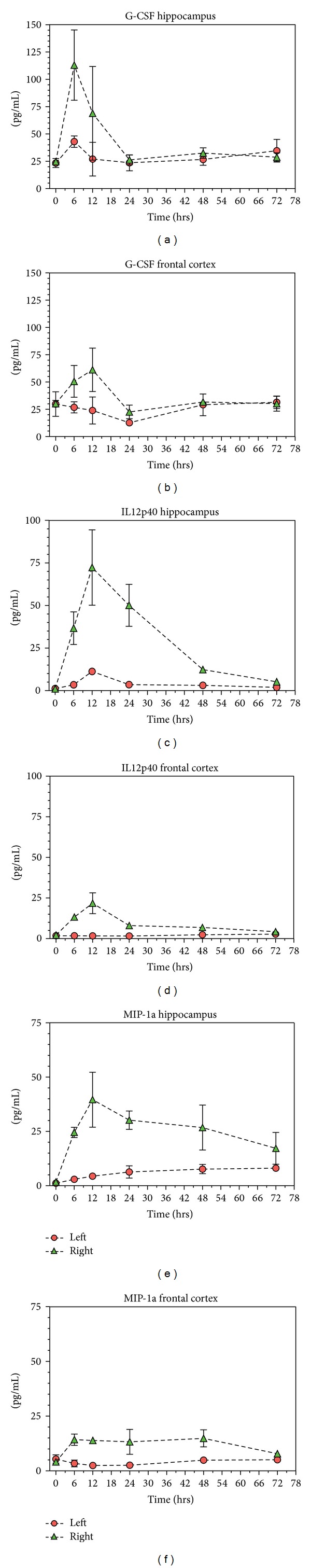
Three of the 17 cytokines measured in hippocampus and frontal cortex (path of the needle track) were significantly changed on the lesioned side compared to the control side. Each time point was determined from *n* = 3 mice (total of 18 pairs of hippocampi), and assays were run in triplicate. 2-way ANOVA (treatment versus time) revealed a significant effect (*P* < 0.05) for each of the cytokines except for G-CSF levels in frontal cortex.

 Within 6 hrs of creating the microlesion, 3 out of 17 soluble cytokines were significantly increased in hippocampus and frontal cortex (along the path of needle insertion). Granulocyte-colony stimulating factor (G-CSF), MIP-1a, and IL12p40 were increased in both hippocampus and frontal cortex (Figure 7). G-CSF levels peaked at 6 hrs after placement of the lesion and returned to levels measured on the unlesioned control side by 24 hrs. IL12p40 concentrations peaked at 12 hrs and were back to baseline by 72 hrs. MIP-1a peaked at 12 hrs and remained elevated until 72 hrs.

## 4. Discussion

 Simple insertion and immediate removal of a sterile fine needle into the dorsal hippocampus triggered a robust cellular response characterized by proliferation of microglia and astrocytes. The microgliosis and astrocytosis remained prominent up to 4 wks, though it declined from the maximum intensity at 2 wks after the lesion. Of the total microglial signal in hippocampus, approximately 36% of these cells were born during the 3-day period after the lesion placement (indicated by double-labeled BrdU-Iba1 cells). The contribution of blood-born cells (indicated by GFP+ cells in chimeric mice) to total microglial burden was approximately 26%. The contribution of blood monocytes to the brain population of microglia is dynamic and can change dramatically following injury, infection, or neurodegenerative processes [24–29]. As shown here, even a brief microinjury as represented by insertion and immediate removal of a fine sterile needle into brain triggers a significant infiltration and activation of blood-derived microglia.

 A potentially beneficial consequence of the microlesion was the stimulation of neurogenesis in the subgranular zone of the dentate gyrus, evidenced by the significant increase in total DCX signal, a marker of immature neurons [30]. The DCX signal at 4 wks remained elevated even as the numbers of double-labeled BrdU-DCX decreased by 4 weeks. The discrepancy between total DCX and BrdU-DCX labeled cells remained high at 4 wks, but the decrease in double-labeled BrdU-DCX at 4 weeks may be explained by (a) programmed cell death of new neurons born during the immediate postlesion period and (b) continued neurogenesis in the period after BrdU injections. The contribution of blood-derived cells (GFP+) to neurogenesis in chimeric mice, as indicated by GFP-Nestin coexpression in subgranular zone, was negligible, suggesting that the source of cells for neurogenesis was within the neurogenic niche itself rather than recruitment of exogenous cells. It is notable that these GFP-Nestin expressing cells did not exhibit radial fibers typical of neural progenitors in the subgranular zone [23]. Nestin-GFP+ coexpression may also indicate development of endothelial cells from bone marrow-derived GFP+ cells.

 The cascade of events that resulted in these cellular responses is complex, but the findings here identify a few salient cytokines that may contribute to the cellular mobilization. Macrophage inflammatory protein-1a (MIP-1a, also known as CCL-3) is known for its chemotactic and proinflammatory effects. Levels of MIP-1a remained elevated for 3 days and most likely played a role in the activation of microglia and astrocytes and also in the recruitment of blood-born monocytes to the site of injury. IL12-p40 (also known as cytotoxic lymphocyte maturation factor 2) is a proinflammatory cytokine with immunoregulatory properties, especially in promoting Th1 cell-mediated immune responses [31]. G-CSF is a hematopoietic cytokine that increases proliferation of blood stem stems and results in increased number of polymorphonuclear leukocytes [32]. More recently, it has been recognized as a neurotrophic factor with antiapoptotic effects and direct actions to promote neurogenesis [33]. In the present study, elevated G-CSF levels may have contributed to hippocampal neurogenesis. Other cytokines were measured (including EGF, BDNF, and various pro- and anti-inflammatory cytokines) but were not found to be significantly altered. A limitation in the present study is that immediate cellular responses to microinjury (hours to several days) were not studied, and so the relationship of the acute cytokine release profile to the immediate cellular response pattern is not available. Nevertheless, the cellular responses documented here at 2 and 4 wks can be seen as a consequence of the acute humoral reaction to the microlesion. More mechanistic studies in the future will be designed to determine the effects of blocking specific cytokines on the cellular responses.

 These findings may be relevant to the growing clinical practice of DBS through chronically implanted metal electrodes into specific brain regions. Electrical stimulation is not required to see signs of neuroinflammation; inflammatory changes have been observed around recording electrodes used for characterizing epileptogenic tissue and around CSF fluid shunt catheters [16, 17]. The animal literature also reveals similar activation of microglia and astrocytes following insertion of electrodes and other intracerebral implants [34–38]. Recently, a study of electrode implantation, without electrical stimulation, has revealed persistent and widespread neuroinflammation in rats, which extends beyond the electrode track in a region-selective manner [39]. Widespread neuroinflammation appears to be a general feature of the chronic implantation procedure, since it was found in rats implanted with three different types of electrodes varying in thickness and shape. 

On the other hand, the enhanced hippocampal neurogenesis elicited by microlesions in young adult mice may not be completely applicable to human patients who are typically older and suffer from conditions such as AD, in which neurogenesis is impaired. However, research with transgenic mouse models of AD (tg APP/PS1) has revealed that the hippocampus retains competency to generate new neurons, especially when triggered by administration of G-CSF or when mice are provided enriched environments and exercise [18, 40].

## 5. Conclusions

 Microinjury was produced by insertion and removal of a fine needle targeting the hippocampus on one side. The lesion caused a time-dependent increase in levels of several inflammatory and anti-inflammatory cytokines. Subsequent histological analysis at 2 and 4 weeks revealed microgliosis and astrocytosis. Microgliosis was a prominent cellular response, and though bone marrow-derived cells contributed to this population of cells, the majority of activated microglia were endogenous to the brain. The microlesion also increased hippocampal neurogenesis, indicated by the increased numbers of immature neurons (DCX+ cells) counted in the sub-granular zone. Based on what is known in the literature about the cytokines (MIP-1a, IL12-p40, and G-CSF), their increased levels very likely contributed to the cellular inflammatory response around and distant from the lesion. These findings are relevant to the growing clinical practice of DBS through chronically implanted metal electrodes into specific brain regions. Electrical stimulation is not required to see signs of neuroinflammation. G-CSF, which has neuromodulatory effects, has previously been shown to increase hippocampal neurogenesis in mice models of Alzheimer's disease, and this correlated with improved performance in a hippocampal-dependent learning task [41]. G-CSF is increasingly recognized as a neurotrophic factor that attenuates neuronal death and enhances functional recovery in various animal models of neurological disorders and is being explored in clinical trials [33, 42–45].
